# Case report: Use of peripheral nerve stimulation for treatment of pain from vertebral plana fracture

**DOI:** 10.3389/fpain.2022.1088097

**Published:** 2023-01-12

**Authors:** Saba Javed, Kenneth Perry, Steven Mach, Billy Huh

**Affiliations:** ^1^ Department of Pain Medicine, University of Texas MD Anderson Cancer Center, Houston, United States; ^2^ Department of Anesthesiology, Critical Care and Pain Medicine, University of Texas Health Science Center at Houston, Houston, United States

**Keywords:** peripheral nerve stimulation (PNS), compression fracture, vertebral fracture, pain, cancer pain, minimally in vasive therapy, multiple myeloma, pain managemant

## Abstract

Vertebral plana fractures are a severe form of compression fractures that can cause significant morbidity due to incapacitating pain. Due to the flattening of the vertebrae in a plana fracture, accessing the vertebral body transpedicularly can be difficult, making traditional vertebral augmentation treatment dangerous. These injuries also typically occur in elderly patients with contraindications to invasive procedures. Peripheral nerve stimulation is a relatively new and minimally invasive treatment that uses electrical stimulation to inhibit pain signals from reaching the somatosensory cortex. Our case describes an 80 Year old female with multiple comorbidities and refractory pain due to a vertebral planar fracture successfully treated with a 60 day course of peripheral nerve stimulation as evidenced by over 50% reduction in symptoms and discontinuation of opioid pain medication use.

## Introduction

Vertebral compression fractures are a prominent medical ailment, with an estimated 1.5 million occurrences each year in the US. Patients with these fractures have been found to use their primary care providers at a rate 14 times higher than the average person within the first year of the injury (1, 2). Fractures often cause incapacitating pain leading to significant morbidity and mortality through reduced physical activity, loss of independence, difficulty sleeping, and decreased lung capacity (2). Although most often caused by osteoporosis, other etiologies such as trauma, cancer, and infection can be culprits (3, 4).

Approximately 75% of compression fractures occur in the thoracolumbar region, ranging from the T12-L2 vertebrae. This is likely due to the region being the transition zone from the rigid thoracic vertebra to the more mobile lumbar vertebrae, creating excess strain on the spine. Utilizing radiography, compression fractures can be classified by the segment of the vertebra that is most affected (4). A vertebral plana or “pancake” fracture is a specific type of vertebral compression fracture defined by a greater than 70% loss of the anterior vertebral height compared to the posterior vertebral height. This can create unique difficulties when it comes to treatment (5, 6).

Initial treatment of vertebral compression fractures typically involves a combination of invasive and non-invasive options targeted toward pain relief and restoration of function, as shown in Table 1. Options include bed rest, nonsteroidal anti-inflammatory medications, narcotics, lidocaine patches, and muscle relaxants. Physical therapy can be considered for patients who are able to tolerate it (7). Facet joint injections, most commonly used for vertebral facet pain, have also been used to treat compression fracture pain as well as other types of low back pain after conservative treatment has failed. Other minimally invasive approaches include radiofrequency ablations (RFAs) and epidural steroid injections (ESI). All three of these techniques involve the use of fluoroscopic guidance to perform nerve blocks in the affected area (8–11).

**Table 1:** Current treatment options for pain due to vertebral compression fracture.

Conservative treatment	Minimally invasive injections	Advanced spine procedure
Bed Rest	Facet joint injections	Vertebroplasty
Physical Therapy	Radiofrequency ablations	Kyphoplasty
NSAIDs	Epidural steroid injections	
Muscle relaxants	Radicular injections	
Narcotics	Peripheral nerve stimulation	

When non-invasive methods do not provide definitive improvement, treatment can be escalated. Moderately invasive options include vertebroplasty or kyphoplasty, both of which involve cement augmentation of the fractured bone. Vertebroplasty is performed by the injection of polymethylmethacrylate (bone cement) through a needle into the vertebral body fracture. When performed percutaneously, it involves accessing the vertebral body through the spinal pedicle. Kyphoplasty is performed similarly; however, it utilizes the insertion of a balloon to expand the height of the vertebral body before the injection of the bone cement (1, 12).

Unfortunately, for patients suffering from vertebral plana fracture, treatment with vertebroplasty is generally not recommended due to complications that can arise from difficulty accessing the vertebral body transpedicularly. One such complication is the extravasation of bone cement into the spinal canal and subsequent compression of the spinal cord (1). Another potential complication is an increased risk of fracture at the vertebral levels above and below the level at which augmentation is performed. Furthermore, the data regarding the effectiveness of vertebral augmentation for pain control conflicts with limited studies showing evidence of pain relief lasting between 1 month to 3 years (2, 12). These fractures largely impact elderly patients who are often not ideal candidates for anesthesia, highlighting a need for alternative and less invasive treatment modalities to treat intractable pain (1, 6).

With these reasons in mind, we are presenting a case utilizing the relatively new concept of non-invasive peripheral nerve stimulation (PNS). PNS is currently FDA-approved for use in intractable chronic back pain, post-surgical pain, and acute pain due to trauma. However, it also has been used to treat facial pain, headaches, phantom limb pain, osteoarthritis, back pain, and cancer pain (13–16). Mechanism of action involves direct stimulation of a specific peripheral nerve *via* electrical impulses. This is a practical application of what is known as the gate control theory. Non-noxious stimulation of AB fibers conveys inhibitory signals to the interneurons present inside the dorsal horn of the spinal cord. In essence, the stimulus closes the “gate” and transmission of pain signals is inhibited from reaching the somatosensory cortex through the spinal cord (17).

Our case examines an 80-year-old woman with multiple comorbidities whose pain due to a vertebral plana fracture was successfully treated with the use of a peripheral nerve stimulator.

## Case

Our patient is an 80-year-old woman with a history of hypertension, hyperlipidemia, chronic obstructive pulmonary disease, resolved possible gastrointestinal bleeding, hypothyroidism, chronic hyponatremia, and a history of squamous cell carcinoma of the tongue with planned glossectomy. She also has a complex cardiac history including a history of sudden cardiac death (over 20 years ago), carotid stenosis status post carotid endarterectomy, and coronary artery disease status post percutaneous coronary interventional with stent placement in 1996. She attended the pain clinic for acute on chronic low back pain. The patient endured a recent fall at home and subsequent worsening of her low back pain, described as axial pain, located predominantly in the lower back, not radiating down the legs. Pain characteristics were described as constant, dull, and achy, and were scaled 9 out of 10 in intensity. It was also noted that the pain worsened with any movement including getting out of bed or out of her chair. The best pain alleviation at this point was to remain sitting in her recliner. At the initial visit, the patient was using diazepam for anxiety, and acetaminophen 650 mg PO TID PRN for pain, which was not effective. She was not taking any other medications for osteoporosis at her initial visit. Her physical exam was limited due to the above-described discomfort; however, the patient was severely tender to palpation in the lumbar paraspinal region, bilaterally, and with positive lumbar facet loading bilaterally.

**Figure 1:**
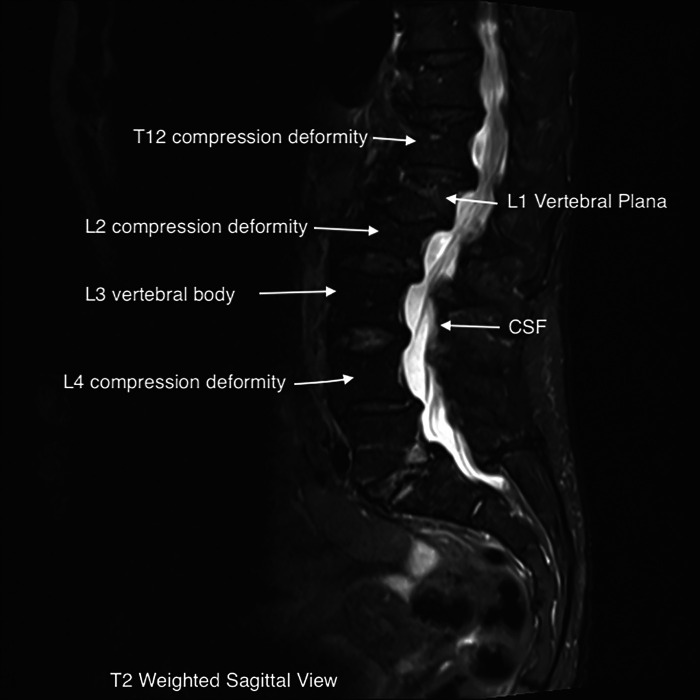
T2 weighted sagittal view.

The patient underwent a lumbar MRI, as shown in Figure 1, which revealed metastatic lesions in the lumbar spine, T12-L2, and L4 compression deformities. The most significant involvement was seen at L1, with near complete vertebral plana (as shown in the figure). Imaging also showed degenerative disc changes at multiple levels.

She was initially started on acetaminophen-codeine 300–30 mg PO TID PRN for severe pain, and was educated on lumbar facet steroid injections, which she rejected because “she did not want steroids”. She was presented with vertebral augmentation, which she rejected for not wanting to undergo a procedure requiring any systemic anesthesia, due to a prior history of sudden cardiac death. Radiofrequency ablation was also discussed; however, the patient declined because she did not want to wait 4 weeks to undergo the two trials of medial nerve branch blocks necessary for insurance to cover the cost of the RFA. Lastly, peripheral nerve stimulation was discussed with the patient, to be done under local anesthesia. All questions were answered, and the patient elected to proceed. The total time between symptom onset and the decision to proceed with PNS placement was approximately 3 weeks.

Two electrode peripheral nerve stimulators (PNS) with SPRINT® were placed at L3 bilaterally to stimulate the multifidi muscles. An external pulse generator (EPG) was connected to the lead to confirm proper placement of the electrode *via* elicited muscle contraction per manufacturer guidelines, i.e., ramped waveform, applied at a frequency of 12 Hz, amplitude (0.1 to 30 mA) and pulse duration (10 to 200µs) were applied intraoperatively (fast-cycle alternating 1-second on, 1-second off) and titrated to patient comfort post-procedure (20-second slow-cycling). After the electrode placement, the patient scaled her pain as 7/10 intensity. One week post-procedure, the patient endorsed improvement in her pain which was scaled at 5/10 and accompanied by a decreased use of acetaminophen-codeine to once a day. At the 1-month and 2-month follow-ups, pain scores were reported at 4/10 and 3/10 respectively, with no usage of acetaminophen-codeine. PNS electrodes were removed after 60 days, per FDA guidance. At the 3-month follow-up after electrode removal, she continued to have pain scored at 3/10 and reported continued cessation of pain medication.

## Discussion

Vertebral compression fractures are a common occurrence in the elderly. Plana fractures are a severe form of these injuries with unique treatment obstacles. For patients that fail conservative treatment, vertebral augmentation has traditionally been the next step in management. Although relatively safe, both vertebroplasty and kyphoplasty procedures carry the risk of complications, including leakage of bone cement, recurrent pain, and fracture of adjacent vertebrae (18).

The type of pain experienced by the patient can vary based on the location of the spinal column at which the pathologic process is occurring. Pain originating from the anterior portion of the spine is typically caused by vertebral body fracture or intervertebral disc degeneration. It is referred to as axial or mechanical pain and is generally localized to the area of injury. Pain is worsened with axial loading in spinal flexion and prolonged standing. Conversely, it is improved with spinal extension and lying supine (19, 20). Pathology in the middle portion of the spinal column is most often due to direct pressure on the spinal cord/peripheral nerve roots as they exit the spinal column. The most common causes include disc herniation, osteophyte formation, or spinal cord stenosis. This type of pain is called radicular pain as it is characterized by radiation of the painful sensation down either leg (20). Finally, pain due to degeneration of the synovium due to osteoarthritis of the facet joints in the posterior aspect of the spinal column is known as facetogenic pain. This accounts for up to 45% of low back pain and is associated with mostly localized discomfort. However, it can sometimes present with symptoms similar to radicular pain in what is referred to as pseudo-radicular pain when synovial cysts form and compress nerve roots as they exit the spinal foramen. Facet loading, which involves simultaneous extension and rotation of the spine, can be a key provocative test (19–21).

The near complete flattening of the vertebrae in a plana facture makes percutaneous treatment with vertebroplasty or kyphoplasty not possible, as was the case in our patient (1). These fractures frequently show weakness or fracture of the posterior wall on imaging. This greatly increases the risk of extravasation of bone cement into the vertebral canal when performing the procedure. In any compression fracture, bony retropulsion into the spinal canal is an absolute contraindication to percutaneous treatment. As exhibited in the images above, our patient's fracture would have made it nearly impossible to safely perform percutaneous intervention and would have required a significantly invasive open procedure (6, 18).

**Figure 2:**
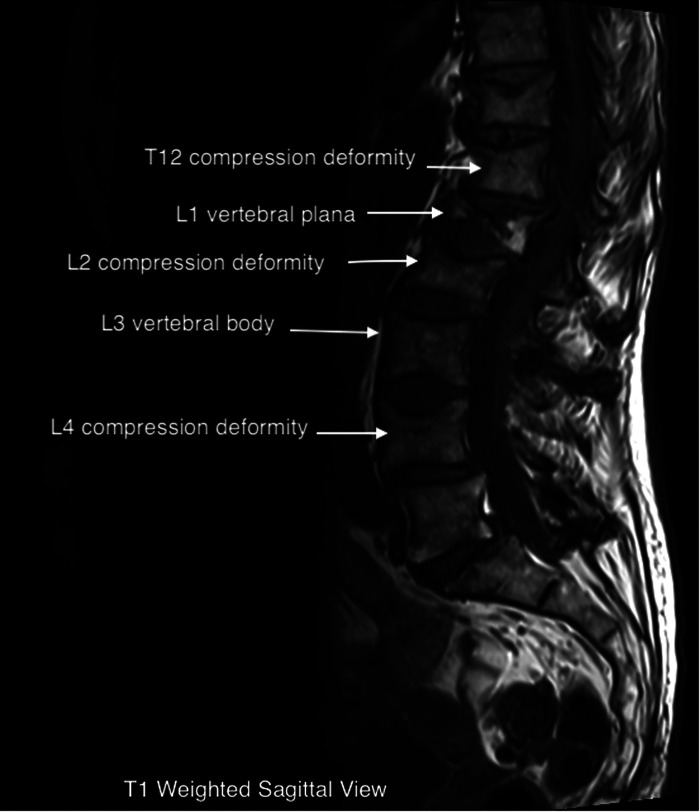
T1 weighted sagittal view.

MRI imaging can be helpful in evaluating the age of the fracture, as new injuries will show areas of hyperintensity on T2 imaging (22). This can be visualized on our patient's L1 plana (Figures 1, 2). Determining the age of the fracture is important, as studies have shown that vertebroplasty is most effective when used to treat fractures less than 6 months old. The definition of an acute compression fracture can vary: typically, they are not considered chronic until after at least 4 months (23).

The ability to lay flat in a prone position and tolerate general anesthesia is required in order to undergo vertebral augmentation as well as RFAs and facet injections. Elderly and frail patients are often poor candidates for anesthesia due to cardiac or pulmonary pathologies (1, 24). Another moderately invasive procedure requiring general anesthesia is spinal cord stimulation (SCS). This has been used successfully to treat back pain of several different causes. SCS was used in a study of 300 patients in treating multifactorial chronic pain in failed back surgery syndrome, vertebral compression fracture pain, and diabetic neuropathy (25). PNS may therefore offer a less invasive replacement for both vertebral augmentation and spinal cord stimulation for patients not able to tolerate anesthesia or sedation.

Pain is typically at its worst during the first few weeks of the fracture, which is therefore when patients are at the greatest risk of debilitation (3, 23). Compression fractures in the thoracoabdominal region often cause severe enough pain to subject patients to prolonged bed rest. This increases the risk of other complications, including constipation, urinary tract infections, deep vein thrombosis, and diminished ability to function independently (26). Additionally, restrictive bracing treatment has been associated with a higher risk of deconditioning, including atrophy of the paraspinal muscles (24). As discussed in our case, the patient's pain was exacerbated by any type of movement. Her pain was described as 9/10 at its worst which prevented her from moving from her chair for most of the day. Three months after treatment her pain was reported as 3/10 and she was able to participate in PT. PNS may serve as a bridge treatment through acute fracture pain after conservative management has failed. The patient also ceased the use of Tylenol with codeine after PNS treatment, which highlights the possibility of PNS being used as part of an opioid-sparing multimodal approach to lower back pain caused by compression fractures.

In addition to the gate control mechanism mentioned above, PNS action in the CNS may be modulated by elevated levels of dopamine and serotonin as well as changes in GABAergic and glycine pathways. These changes in neurotransmitter quantities have shown some evidence of gene transcription alterations in rat studies as well as the enhancement of NMDA receptor plasticity. Such alterations may give credence to long-lasting pain control after treatment has been stopped. Research also has shown that repetitive stimulation of A-Delta and C fibers can reduce the excitation of these fibers over time, creating a localized peripheral decrease in pain which may help provide longer-lasting pain relief even after treatment has been completed (17).

A review of the literature regarding PNS treatment revealed a randomized controlled trial that was successful in relieving peripheral nerve pain with greater than 50% efficacy 3 months after treatment. Evidence includes trials showing efficacy in the treatment of pelvic and cluster headaches (27). Case series data exhibited successful implementation of PNS to treat oncologic pain, with success defined as a greater than 50% reduction of pain. Three of these cases involved the treatment of spinal lumbar and thoracic pain. Other studies have shown the effectiveness of PNS in treating extremity pain by targeting the brachial plexus and other peripheral nerves (13).

According to Medicare data, the average cost of peripheral nerve stimulator placement in an ambulatory surgery center is around $5,000, which is fully reimbursable. In comparison, the cost of vertebroplasty and kyphoplasty can range from $10,000 to $15,000. Epidural steroid injections can range from $500-$1000 when paid for by Medicare (28, 29).

However, with PNS being a relatively new treatment, there is little literature and no case studies found on its use in successfully treating pain specifically due to vertebral plana fracture.

## Conclusion

Vertebral plana fractures refractory to conservative treatment are difficult to treat with current, minimally invasive techniques due to their anatomical configuration. The occurrence and greater prominence in elderly patients who may not tolerate anesthesia or vertebral augmentation increase treatment complexity. In the case of the patient, symptoms decreased by 4 points on the pain scale, she discontinued taking opioid pain medication and was able to participate in PT. PNS has been effectively used to treat several types of pain and more research is needed into its effectiveness in treating refractory pain due to vertebral plana fracture.

## Data Availability

The original contributions presented in the study are included in the article/Supplementary Material, further inquiries can be directed to the corresponding author.
